# Annealing-modulated nanoscintillators for nonconventional X-ray activation of comprehensive photodynamic effects in deep cancer theranostics

**DOI:** 10.7150/thno.41752

**Published:** 2020-05-20

**Authors:** Yao-Chen Chuang, Chia-Hui Chu, Shih-Hsun Cheng, Lun-De Liao, Tsung-Sheng Chu, Nai-Tzu Chen, Arthur Paldino, Yu Hsia, Chin-Tu Chen, Leu-Wei Lo

**Affiliations:** 1 Institute of Biomedical Engineering and Nanomedicine Research, National Health Research Institutes, 35 Keyan Road, Zhunan 350, Taiwan.; 2 Department of Radiology, The University of Chicago, Chicago, IL 60637, USA.; 3 Department of Cosmeceutics, China Medical University, 91 Hsueh-Shih Road, Taichung, Taiwan 40402.; 4 Faculté de médecine Lyon-Est, Université Claude Bernard, 8 Avenue Rockefeller, Lyon 69008, France.

**Keywords:** Photodynamic therapy, annealing, nanoscintillator, fractionated radiotherapy, X-ray induced radioluminescence, vascular remodeling, radioresistance

## Abstract

Photodynamic therapy (PDT), which involves the generation of reactive oxygen species (ROS) through interactions of a photosensitizer (PS) with light and oxygen, has been applied in oncology. Over the years, PDT techniques have been developed for the treatment of deep-seated cancers. However, (1) the tissue penetration limitation of excitation photon, (2) suppressed efficiency of PS due to multiple energy transfers, and (3) insufficient oxygen source in hypoxic tumor microenvironment still constitute major challenges facing the clinical application of PDT for achieving effective treatment. We present herein a PS-independent, ionizing radiation-induced PDT agent composed of yttrium oxide nanoscintillators core and silica shell (Y_2_O_3_:Eu@SiO_2_) with an annealing process. Our results revealed that annealed Y_2_O_3_:Eu@SiO_2_ could directly induce comprehensive photodynamic effects under X-ray irradiation without the presence of PS molecules. The crystallinity of Y_2_O_3_:Eu@SiO_2_ was demonstrated to enable the generation of electron-hole (e^-^-h^+^) pairs in Y_2_O_3_ under ionizing irradiation, giving rise to the formation of ROS including superoxide, hydroxyl radical and singlet oxygen. In particular, combining Y_2_O_3_:Eu@SiO_2_ with fractionated radiation therapy increased radio-resistant tumor cell damage. Furthermore, photoacoustic imaging of tumors showed re-distribution of oxygen saturation (_S_O_2_) and reoxygenation of the hypoxia region. The results of this study support applicability of the integration of fractionated radiation therapy with Y_2_O_3_:Eu@SiO_2_, achieving synchronously in-depth and oxygen-insensitive X-ray PDT. Furthermore, we demonstrate Y_2_O_3_:Eu@SiO_2_ exhibited radioluminescence (RL) under X-ray irradiation and observed the virtually linear correlation between X-ray-induced radioluminescence (X-RL) and the Y_2_O_3_:Eu@SiO_2_ concentration *in vivo*. With the pronounced X-RL for *in-vivo* imaging and dosimetry, it possesses significant potential for utilization as a precision theranostics producing highly efficient X-ray PDT for deep-seated tumors.

## Introduction

More than ten million individuals worldwide are diagnosed with cancer annually, and effective technologies for eradicating tumors are urgently needed. Traditional approaches such as surgery, chemotherapy, radiation therapy, photodynamic therapy (PDT), and hyperthermia have shown great success in widespread clinical and preclinical use. Among those promising approaches, PDT, a noninvasive and site-directed medical technique based on the destruction of tumor tissues by light inducing reactive oxygen species (ROS) generation, has undergone extensive investigations in recent years. In PDT, a photosensitizer (PS) is activated by a specific wavelength of light to generate ROS or free radicals that can react with the local microenvironment, and eventually results in cell death and tissue devastation 1,2. In terms of spatial selectivity in PDT, specific targeting of the PS to the tumor compartment by attaching with various targeting ligands is crucial. Tumor specific accumulation followed by local irradiation facilitates targeted damage to malignant tissue while sparing surrounding healthy tissues 3.

To date, the current clinical use of PDT has been limited to superficial layers of tissues, such as in skin cancer, lung cancer, and esophageal cancer that are easily accessible. A major challenge in most traditional PDT is the difficulty in delivering UV or visible light into the subsurface for PS activation. This limits clinical applications of PDT due to the short penetration depth of illumination light, which leads to ineffective treatment of deep-seated tumors 4,5. One auspicious strategy to overcome the limitation of PDT for deep tumor treatment is the development of novel near-infrared (NIR) PSs or using nanoparticles as PS carriers to establish two-photon PDT or upconversion nanoparticle- mediated PDT 6-9, which aims to minimize tissue interference and improve penetration depth. For example, Cheng et al. showed that the mesoporous silica nanoparticles (MSN) co-encapsulated two-photon absorbing dyes and PS enabled high-energy transfer rates for two-photon activated PDT. The two-photon PDT system enhanced the energy transfer rate up to unprecedented 93% to yield efficacy in deep tissue 10. In another study by Chen et al., plasmon-enhanced photoluminescence of GNRs induced by two-photon excitation at NIR wavelength demonstrated the intra-particle energy transfer. This phenomenon induced singlet oxygen generation, which is worthy of studying its potential for development in clinical translation 11. However, even with this advancement, the photon penetration depth can only be increased to a few centimeters into soft tissue 12. Moreover, reduced ^1^O_2_ generation efficiency has been reported by using NIR-activated PSs, and the efficiency of energy transfer from NP toward the PS has usually limited the clinical use of PDT 8,13.

Many recent alternative approaches have been developed to improve tissue penetration capability of PDT, including microwave 14,15, ultrasound 16 and X-ray 17-19. Among them, X-ray, with the highest penetration depth, was proposed as an energy source to interact with scintillating nanoparticles and then activate PDT, which could be termed as X-ray excited PDT (hereinafter called as X-ray PDT). In a pilot study, Chen et al. developed self-lighting PDT, in which nanoparticles upon exposure to extrinsic X-rays emit luminescence in the visible region of the spectrum, activating the attached PS, and resulting in the efficient generation of ^1^O_2_ 20. Apart from this, the use of photons from Cerenkov radiation (CR) has been emerging as an internal light source to activate PS *in vivo*. Cerenkov luminescence is mainly composed of UV-blue photons, which deliver rapid and localized excitation source for Cerenkov luminescence induced PDT without prolonged radiation exposure to healthy tissue 21. Even though, remarkable results of cancer eradication *in vitro* and *in vivo* have been observed in recent years 22-24, the investigation, evaluation and application of deep PDT for clinical use are still in its infancy. Much room remains for improving the efficiency of X-ray PDT, including photobleaching of organic PSs during X-ray irradiation 25, PS loading efficiency for maximized ^1^O_2_ production 24,26, matching and fluorescence resonance energy transfer (FRET) efficiency between scintillator and PS 27,28, and illumination fluence and fluence rate 29,30. Moreover, the hypoxia microenvironment of tumor, either pre-existing or as a result of oxygen depletion during PDT, can significantly decrease the effectiveness of PDT-induced cell killing 8,31,32. To minimize the hypoxia-limited therapeutic effect in PDT, a circumventive strategy was developed involving a combination of scintillators and inorganic PSs, such as ZnO or TiO_2_. These approaches not only reduced the possibility of PS photodestruction, but also provided a solution to diminish the O_2_-dependence of PDT 33. Furthermore, in the context of cancer therapy, radiation therapy still plays a critical role in the management of more than 50% of cancer cases. The success of radiation therapy also depends on clinical and radiobiological factors. For example, delivering the dose in single or hypofractionated schedule may increase the therapeutic index of treatment compared to conventionally hyperfractionated radiation therapy. However, hyperfractionated radiation therapy could open temporarily closed blood vessels and result in as short-term local reoxygenation and change cellular radiosensitivity during multifraction delivery 34. Therefore, the radiation dose is believed to be a double-edged sword in X-ray PDT.

Herein, we reported the development of silica-coated scintillating Y_2_O_3_:Eu nanoparticles (Y_2_O_3_:Eu@SiO_2_) that undergo annealing treatment could exhibit not only enhanced photoluminescence efficiency, but also the ability to generate cytotoxic singlet oxygen, superoxide anion and hydroxyl radical upon X-ray irradiation without the incorporation of any additional PS. In this case, the excitation energy is absorbed first by the host lattices, and the electrons (e^-^) of NPs are promoted across the band gap to the conduction band, which creates a hole (h^+^) in the valence band. We observed that ROS generation may result from electrons in the conduction band and holes in the valence band that exhibit high reducing and oxidizing power, respectively. We further implemented a fractionated radiation regimen with Y_2_O_3_:Eu@SiO_2_ to explore an approach relevant to clinical conventionally fractionated radiation therapy. By comparing radio-sensitive (CAOV3) and radio-resistant (SKOV3) ovarian cancer cells, our established annealing-modulated Y_2_O_3_:Eu@SiO_2_ could improve the radiotherapy efficacy of CAOV3 cells by means of ROS generation and inducing reoxygenation to overcome tumor hypoxia. Our work demonstrated that the as-synthesized Y_2_O_3_:Eu@SiO_2_ had potential applications in X-ray PDT with RL-visualized nanodosimetry to overcome the radio-resistant cancers.

## Experiment section

### Chemicals and materials

Y(NO_3_)_3_, Eu(NO_3_)_3_, urea, tetraethylorthosilicate (TEOS), ammonium hydroxide (30%), and ethanol were purchased from Acros. Diphenolbenzofuran (DPBF), dihydroethidium (DHE), coumarin-3-carboxylic acid (3-CCA), and 5-tertbutoxycarbonyl-5-methyl-1-pyrroline N-oxide (BMPO) were obtained from Sigma Chemical Co. Propidium Iodide (PI) and YO-PRO-1 was purchased from Invitrogen. All chemicals were used as received without further purification.

### Synthesis of Y_2_O_3_:Eu@SiO_2_ nanoparticles

In a typically synthetic procedure via the urea homogeneous precipitation method 35, 0.04 mol•L^-1^ Y(NO_3_)_3_·6H_2_O, 0.002 mol•L^-1^ Eu(NO_3_)_3_·6H_2_O, and 2 mol•L^-1^ urea were mixed together, and then aged for 4 h at 85 ºC. Then, the light-white precursors were collected by centrifugation, washed repeatedly with deionized water, and then dried in an oven at 60 ºC. The precursor of Y_2_O_3_:Eu was obtained after drying this product at 80 ºC for 12 h. Coating of SiO_2_ was carried out by hydrolysis of tetraethoxysilane (TEOS) 34. Firstly, 250 mg of Y_2_O_3_:Eu NPs was dispersed in 100 mL ethanol by sonication for 15 min, and then mixed with 1 mL ammonia (25 wt% purity) with vigorous stirring. Then 0.6 mL TEOS was then added dropwise to the solution. After stirring at room temperature for 1.5 h, the resultant products were washed several times with ethanol and water, and the as-synthesized materials were dried at 60 ºC, and then sintered at 800 ºC in air for 4 h.

### Characterization

Particle morphology of Y_2_O_3_:Eu and Y_2_O_3_:Eu@SiO_2_ samples was characterized via transmission electron microscope (TEM, Hitachi, H-7650 operating at an acceleration voltage of 80 kV). The excitation sources for the X-ray photoluminescence spectra and X-ray excited optical imaging were the intensity modulated radiotherapy system at the University of Chicago. The irradiator was operating in the range from 40 to 225 kV acceleration potential (no Cu filter), 1~25 mA tube current, and a 15 mm diameter collimator. The emission spectra were recorded using fiber optic cables connected to a fluorescence spectrometer (USB 4000) from Ocean Optics. For the optical imaging, 10 mg of Y_2_O_3_:Eu@SiO_2_ nanoparticles was packed into a 1.6 mm ID borosilicate glass tube phantom. The phantom was irradiated with the 140 kV / 20.89 mA, a 15 mm diameter collimated X-ray source, and imaging was performed by a Canon 5D Mark II with Canon 35 mm lens, F 1.4 L (stopped down to F 8.0).

### Reactive oxygen species (ROS) detection in solution

For the quantification of ROS, three specific species, i.e., singlet oxygen (^1^O_2_), superoxide anion (O_2_^•-^), and hydroxyl radical (•OH), were chosen for their biological importance. Generation of these species was measured by three kinds of probes, DPBF (absorbance: 402 nm) and 3-CCA (excitation: 395 nm, emission: 450 nm) were dedicated to the quantification of integrated amounts of ^1^O_2_, and •OH, respectively. Both of DHE and BMPO were assigned to the quantification of O_2_^•-^ 37-39. Two hundred and fifty micrograms of Y_2_O_3_:Eu@SiO_2_ was suspended with 1 mL PBS for ROS measurement. ROS probe was premixed with Y_2_O_3_:Eu@SiO_2_ and then diluted by PBS for the designed final concentration. The resulting final concentrations of DPBF, DHE, and 3-CCA were 40 μM, 10 μM and 25 μM, respectively. Solutions were then exposed to X-rays by using a commercial cabinet X-ray system with the standard X-ray tube operated at 150 kV and 15 mA. Singlet oxygen measurements were made by following the loss of UV absorbance of DPBF in the aqueous Y_2_O_3_:Eu@SiO_2_ solutions. In the DHE measurement, the solution was excited at 465 nm, and its fluorescence intensity at 585 nm was measured. In the BMPO measurement, the characteristic spectrum of BMPO/•OH adduct (a^N^ = 13.56, a^β^_H_ = 12.30, and a^γ^_H_ = 0.66) was measured.

### Phantom preparation and calibration curve establishment

Intralipid, with a refractive index close to soft tissue and negligible absorption in visible spectral region has been widely used as tissue-mimicking phantom. Intralipid phantoms were prepared according to the literature 38. Briefly, 2.5 g of type A gelatin was added to 50 mL of deionized water, then the mixture was heated to a temperature above the gelatin's melting point until the gelatin dissolved completely and the solution turned transparent. Subsequently, 0.5 g of intralipid and various concentrations of Y_2_O_3_:Eu@SiO_2_ were added into the gelatin-water solution followed by filling cylindrical molds with the final solution, and let the hydrogel solidify at room temperature. For constructing the calibration curve, an imaging system was established; consisting of an X-ray source (RS-2000, Rad Source Co., Ltd) positioned approximately 20 cm above the intralipid phantom, and a CCD camera module placed perpendicularly to the irradiation source and 30 cm away from the phantom. Black curtains were used to darken the imaging space. Acquired X-RL images were further processed using MATLAB.

### *In vivo* radioluminescence imaging

For RL studies, nude mice were anesthetized with isoflurane (1.5~2% inhalation), and the SKOV3 human ovarian cells (5 x10^6^ cells in 0.2 mL PBS) were subcutaneously injected into right flanks of the mice. When the tumors reached approximately 500 mm^3^, tumors were intratumoral injected with different concentration of Y_2_O_3_:Eu@SiO_2_ and imaged after 1 h using the homemade radioluminescence system operating at 160 kVp and 5 mA. At the end of the study when the survived mice were sacrificed, all tumors in each study group were measured by ICP-MS to quantify the concentration of Y_2_O_3_:Eu@SiO_2_. All mice were anesthetized with an intraperitoneal injection of a ketamine hydrochloride and xylazine cocktail prior to imaging.

### Cell culture

SKOV3 (human ovarian cancer) and CAOV3 (human ovarian cancer) cell lines were purchased from American Tissue Culture Collection (ATCC). SKOV3 and CAOV3 cells were cultured in McCoy's 5A (Gibco) and DMEM (Gibco), respectively. All cell lines were incubated at 37 °C in a fully humidified atmosphere of 5% CO_2_.

### *In vitro* confocal images of cytotoxicity of Y_2_O_3_:Eu@SiO_2_ and radiotherapy effects

To determine the *in vitro* radiotherapy effects, SKOV3 and CAOV3 ovarian cancer cells were cultured in McCoy's 5A and DMEM medium containing 10% fetal bovine serum (FBS) at 37 °C under 5% CO_2_. Cells were seeded at a density of 3×10^4^ on a 60 mm dish to permit cell attachment. Until 80% cell density, the cells were carefully washed with phosphate-buffered saline (PBS), refreshed with fresh medium containing 50 μg mL^-1^ of Y_2_O_3_:Eu@SiO_2,_ and then incubated at 37 °C and 5% CO_2_ for 3h or 24 h. After incubation, the cells were rinsed with PBS three times to remove the excess nanoparticles and placed in fresh medium that contained YOPRO-1 and PI dyes. The cell nuclei were stained with 1 μM Hoechst 33342 for 10 min and washed three times with PBS. For cell morphology and fluorescence imaging, YOPRO-1 was excited at 488 nm and monitored using a 525 ± 5 nm bandpass filter, while PI was excited at 535 nm and monitored using a 620 ± 5 nm bandpass filter. Images were collected using a confocal microscope (Zeiss LSM 710).

### Cytotoxicity *in vitro*

The cytotoxic effect of the Y_2_O_3_:Eu@SiO_2_ was determined by a standard MTT assay in 96 well plates. Approximately 5 × 10^3^ cells were placed in each well and cultured at 37 °C with 5% CO_2_. After 24 h of incubation, the media were removed and replaced with fresh medium supplemented with 10% FBS containing Y_2_O_3_:Eu@SiO_2_ at concentrations ranging from 0 μg mL^-1^ to 100 μg mL^-1^. After 24, the medium containing Y_2_O_3_:Eu@SiO_2_ was replaced by 180 μL fresh medium, followed by the addition of 20 μL MTT. After 4 h of culturing, the medium was substituted with DMSO (150 μL); the survival rate was evaluated via absorbance measurements at 570 nm.

### Clonogenic assay

Appropriate cell numbers were plated for survival analysis. Culture medium was removed and replaced with nanoparticle-containing culture medium for 24 h at 37 °C. Radiation was delivered in a single dose of 1~2 Gy over an appropriate field size at 150 keV and 250 keV, respectively. After irradiation, cells were harvested, counted, and seeded on a 100 mm dish. Cell cultures were then incubated for 10-14 days at 37 °C in 5% CO_2_ in air and 95% humidity. Colonies are fixed with glutaraldehyde (6.0% v/v), stained with crystal violet (0.5% w/v), and counted with each experiment performed in quadruplicate.

### *In vivo* radiotherapy combined with X-ray activated PDT effect

All experiments that involved animals were performed in accordance with the guide for the care and use of laboratory animals and approved by the Institutional Animal Care and Use Committee of National Health Research Institutes. *In vivo* experiments were conducted using five to six-week-old female nude mice (BioLASCO Taiwan Co., Ltd.). The mice were anesthetized with isoflurane (1.5~2% inhalation), and the SKOV3 human ovarian cells (5 x10^6^ cells in 0.2 mL PBS) were subcutaneously injected into right flanks of the mice. The tumors were allowed to reach approximately 500 mm^3^ in volume estimated with the formula 1/2 (L × W^2^), where L and W were the length and width, respectively. The mice were randomized to various treatment groups: control (n= 6), Y_2_O_3_:Eu@SiO_2_ (16 mg/kg, n = 6), radiation (total 8 Gy; four fractions of 2 Gy every day, n = 6), and combination (n=6). In the group of combined treatment, 0.1 mL of 16 mg/kg Y_2_O_3_:Eu@SiO_2_ solution was administered to each mouse *via* multipoint intratumoral injection. After 1 h post-injection, the tumors in mice were irradiated using an X-ray irradiator (RS-2000, Rad Source Co., Ltd). Tumor volume observations were measured by caliper twice per week. For each group, immunohistochemical analyses were performed on tumors collected 5 d post-injection and end of experiment.

### Measurement of oxygen distribution by photoacoustic imaging

To measure the oxygen saturation (sO_2_) inside the SKOV3 solid tumor, the PA C-scan (i.e., two-dimensional scanning) was performed to acquire functional and reference images. Before and after intratumoral administration of 1 mg of Y_2_O_3_:Eu@SiO_2_ and treated with 8 Gy X-ray (four fractions of 2 Gy every day), sO_2_ around the tumor was measured at the end of radiation therapy by the differential optical absorption of oxygenated, deoxygenated hemoglobin and total hemoglobin at different wavelengths of 850 nm, 750 nm and 800, respectively. To facilitate comparison of sO_2_ patterns at different groups, the regions of interest (ROI) in tumor were employed in the proximity of reagents' injection site, and identified using ultrasound imaging. Photoacoustic B-scans of the mice generating average oxygenated and deoxygenated hemoglobin signals were analyzed by custom-developed software based on MATLAB® (R2007a, The MathWorks, USA), and sO_2_ is defined as sO_2_ = [HbO_2_]/ [HbO_2_]+[Hb].

### Dual-color immunofluorescent analysis of tumors

The therapeutic effects were determined by active caspase-3 and Annexin V immunostaining. The cryosections of tumor tissue collected from 5 d post-injection (5 μm thick) were washed three times, with PBS for 5 min and fixed with freshly prepared 4% paraformaldehyde in PBS, for 15 min at room temperature. The sections were probed overnight with a polyclonal rabbit anti-mouse active caspase-3 antibody (GeneTex) and rabbit anti-mouse Annexin V (GeneTex) at 4 °C, followed by incubation with peroxidase-conjugated polyclonal goat anti-rabbit antibodies IgG (DyLight488) (GeneTex) and goat anti-rabbit IgG (Alex Fluor594) for 1 h, at room temperature. The sections were counterstained with Hoechst 33342 for 30 min and mounted with glass coverslips. The tissue sections were then analyzed by fluorescence microscopy.

The cryosections of tumor tissue collected from end of experiment (5 μm thick) were fixed with freshly prepared 4% paraformaldehyde in PBS, for 15 min at room temperature and washed 3 times with PBS. The sections were probed overnight with mouse anti-CD31 antibody (GeneTex) and rabbit anti-αSMA antibody (GeneTex) at 4 °C, followed by incubation with polyclonal goat anti-mouse IgG (DyLight488) antibodies and polyclonal goat anti-rabbit IgG (DyLight594) antibodies (GeneTex) for 1 hour, at room temperature. The sections were counterstained with Hoechst 33342 for 30 min and mounted with glass coverslips. The tissue sections were then analyzed by fluorescence microscopy and quantified by ImageJ software.

## Results and discussion

**Figure 1:**
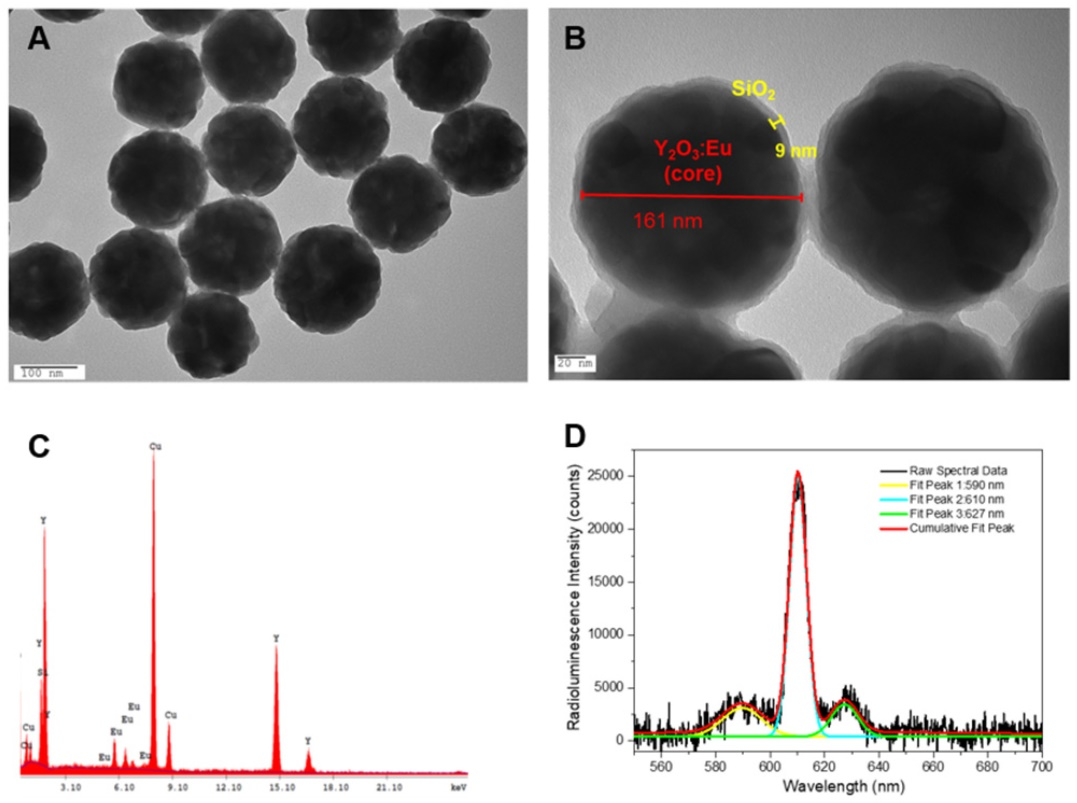
** Characterization of Y_2_O_3_:Eu@SiO_2_. TEM images of** (A) Y_2_O_3_:Eu and (B) Y_2_O_3_:Eu@SiO_2_; (C) EDX analysis of Y_2_O_3_:Eu@SiO_2_ particles, and (D) Typical radioluminescence spectra showing dominant emission peaks at 590, 610 nm, and 627 nm, as well as their corresponding, independent Gaussian curve-fits.

The low-temperature reflux method, utilizing urea-assisted precipitation, was employed to systematically synthesize nanocrystal compositions. The morphology of calcined Y_2_O_3_:Eu was characterized by TEM and is shown **Figure 1A**. From the image, it can be observed that the naked Y_2_O_3_:Eu nanoparticles appeared as homogeneous and monodisperse nanospheres with an average size of 160 nm. For biological applications, water solubility is critical. Coating Y_2_O_3_:Eu with SiO_2_ is the most popular strategy to increase the water solubility of nanoparticles. Additionally, since the induced luminescence could be quenched by water and organic molecules *via* a mechanism similar to fluorescence resonance energy transfer (FRET), SiO_2_ could decrease non-radiative transition to prevents the quenching of luminescence quenching 41,42. Therefore, the Y_2_O_3_:Eu nanoparticles were coated with a thin layer of silica using the modified Stöber method and the core-shell structure for the Y_2_O_3_:Eu@SiO_2_ can be clearly seen due to the different electron penetrability for the cores and shells (as shown in **Figure 1B**). The Y_2_O_3_:Eu cores are black spheres with an average size of 160 nm, and the shells have a gray color with an average thickness of 9 nm. It was reported by Liu and colleagues that silica-based nanoparticles with size between 100 and 200 nm own the best efficiencies of the cellular uptake and endosomal escape 43,44. In addition, the coating thickness of outer silica layer has some effect on ROS generation. Due to the extremely short lifespan and severely limited diffusion distance (< 20 nm), ROS only acts in its immediate vicinity. To strike the balance between ROS generation and biocompatibility, a thin silica-layer (<10 nm) was used in our study. **Figure 1C** shows the EDX spectra of Y_2_O_3_:Eu@SiO_2_. It is explicitly shown from **Figure 1C** that Y, Eu, and Si peaks are at normal energies, which indicates that the composition of coated layer is SiO_2_. The TEM and EDS results as described above confirm the uniform coating of silica shell on our synthesized Y_2_O_3_:Eu@SiO_2_ nanoparticles. Following synthesis, the optical properties of Y_2_O_3_:Eu@SiO_2_ nanoparticles were thoroughly characterized. A typical RL spectrum of an aqueous suspension of Y_2_O_3_:Eu@SiO_2_ nanoparticles under X-ray irradiation is shown in **Figure 1D**. The complete RL spectra were collected and subsequently curve-fitted (Gaussian), and exhibited the characteristic luminescence bands at 590 nm, 610 nm, and 627 nm, which can be assigned to ^5^D_0_ → ^7^F_1_, ^5^D_0_ → ^7^F_2_, and ^5^D_0_ → ^7^F_3_ transitions of the Eu^3+^ ion, respectively, indicating that Eu^3+^ sites act as luminescence centers. Based upon the findings in the extant literature, the spectral properties of our RL spectrum of Y_2_O_3_:Eu@SiO_2_ nanoparticles appear essentially similar to those that were recorded under either UV excitation or X-ray irradiation 45,46.

**Figure 2:**
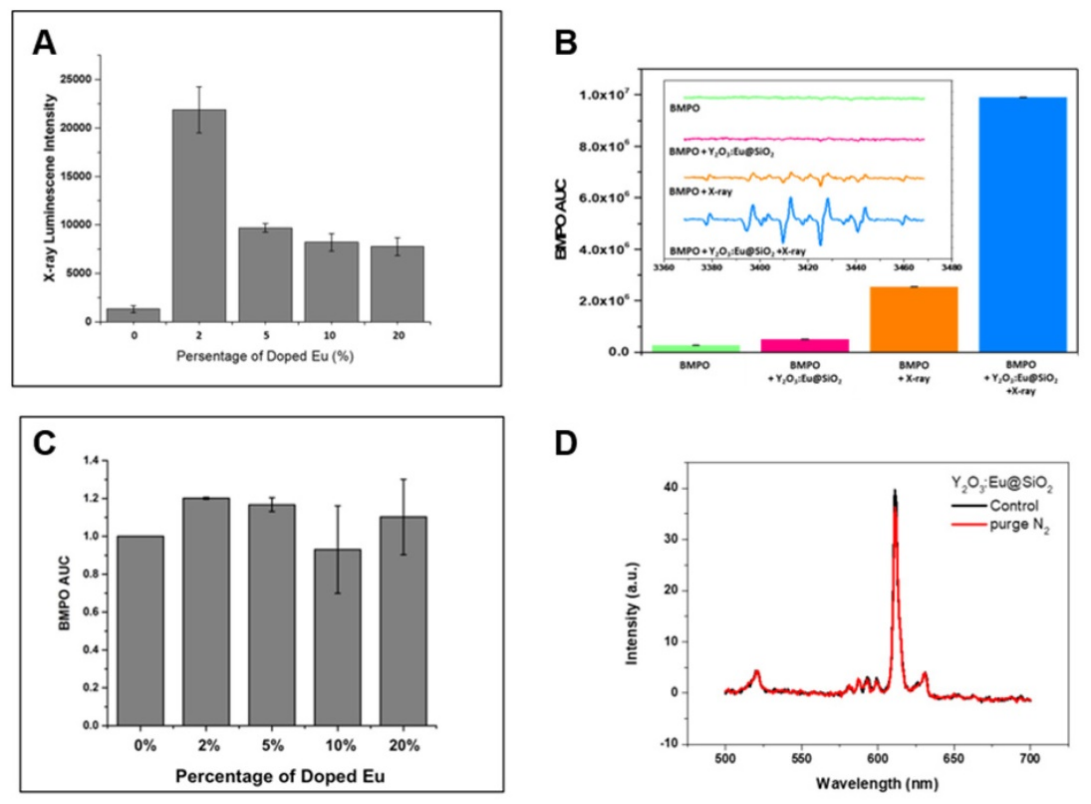
** RL and O_2_^•-^/•OH generation variation at different conditions**. (A) Emission spectrum Y_2_O_3_:Eu@SiO_2_ with different Eu^3+^; (B) concentration demonstration of superoxide anion/hydroxyl radical enhanced by Y_2_O_3_:Eu@SiO_2_ under ionizing irradiation; (C) Relative ESR intensity Y_2_O_3_:Eu@SiO_2_ with different Eu^3+^ concentration. (D) Emission spectra of Y_2_O_3_:Eu@SiO_2_ nanoparticle before (black) and after bubbling N_2_ gas (red).

We have compared photoluminescence and ROS generation efficiency between annealed and pre-annealed nanoparticles. Both photoluminescence and ROS generation of pre-annealed Y_2_O_3_:Eu nanoparticles are significantly weaker than those of annealed Y_2_O_3_:Eu nanoparticles (data no shown), the results are in consistent with previous reports. The precursor (Y(OH)CO_3_·H_2_O):Eu^3+^ nanoparticles contains many hydroxyl groups and is either amorphous or paracrystalline. Due to the defects produced during the preparation process and impurities contained in the raw materials, the samples inevitably have quenching centers (traps), which induce non-radiative transitions and decrease the photoluminescence and ROS generation efficiency. Therefore, a calcination process is indispensable for dehydroxylation and crystallization 47-49. We next investigated X-RL imaging and radio-sensitization of different Eu doping concentrations to determine if there is an Eu-dependent effect. First, the dependence of RL intensity from Eu^3+^ content was measured, as showed in **Figure 2A**. It is observed that the relative intensity of 610 nm X-RL first increased up to 2 mol% of Eu^3+^ concentration, and then decreased while Eu^3+^ dopant concentration continuously increased. This decrease of X-RL as the doping Eu^3+^ level higher than 2 mol % should be resulting from the concentration dependent quenching caused by the cross-relaxation between neighboring Eu^3+^ ions 50,51. Thus, the optimal concentration of Eu^3+^ ions in our prepared Y_2_O_3_:Eu@SiO_2_ is 2 mol% at the time of synthesis. Scintillators are generally capable of converting high-energy X-rays and gamma rays into UV-visible lights. Limited investigations exist on ROS generation from scintillators. In the case of X-ray inducible PDT, energy transfer may occur if a significant overlap exists between the emission of the donor and the absorption of the acceptor. In order to elucidate the ROS generation mechanism in our study, the contribution of Eu^3+^ concentration to the enhanced generation of hydroxyl radical under X-ray irradiation was estimated by electron paramagnetic resonance (EPR) spectrum (**Figure 2B**). In the case in which Y_2_O_3_:Eu@SiO_2_ was mixed with BMPO under X-ray irradiation, the EPR revealed a four-line spectrum which implied an BMPO-OH adduct 52. It is worth noting that as shown in **Figure 2C**, the intensities of O_2_^•-^/•OH EPR signals for the as-prepared particles with various Eu^3+^ doping concentrations showed no significant difference. Thus, we do not expect a dominant contribution of Eu^3+^ ions to the ROS generation process under X-ray excitation. At the same time, the RL of Y_2_O_3_:Eu@SiO_2_ was also observed in the absence of O_2_, as shown in **Figure 2D**. Moreover, no significant RL variation was found between the normal and O_2_ ablation conditions, which indicated that implied RL in our established system is an O_2_- systems, and thus Y_2_O_3_:Eu@SiO_2_ could be employed in image-guided radiotherapy.

**Figure 3:**
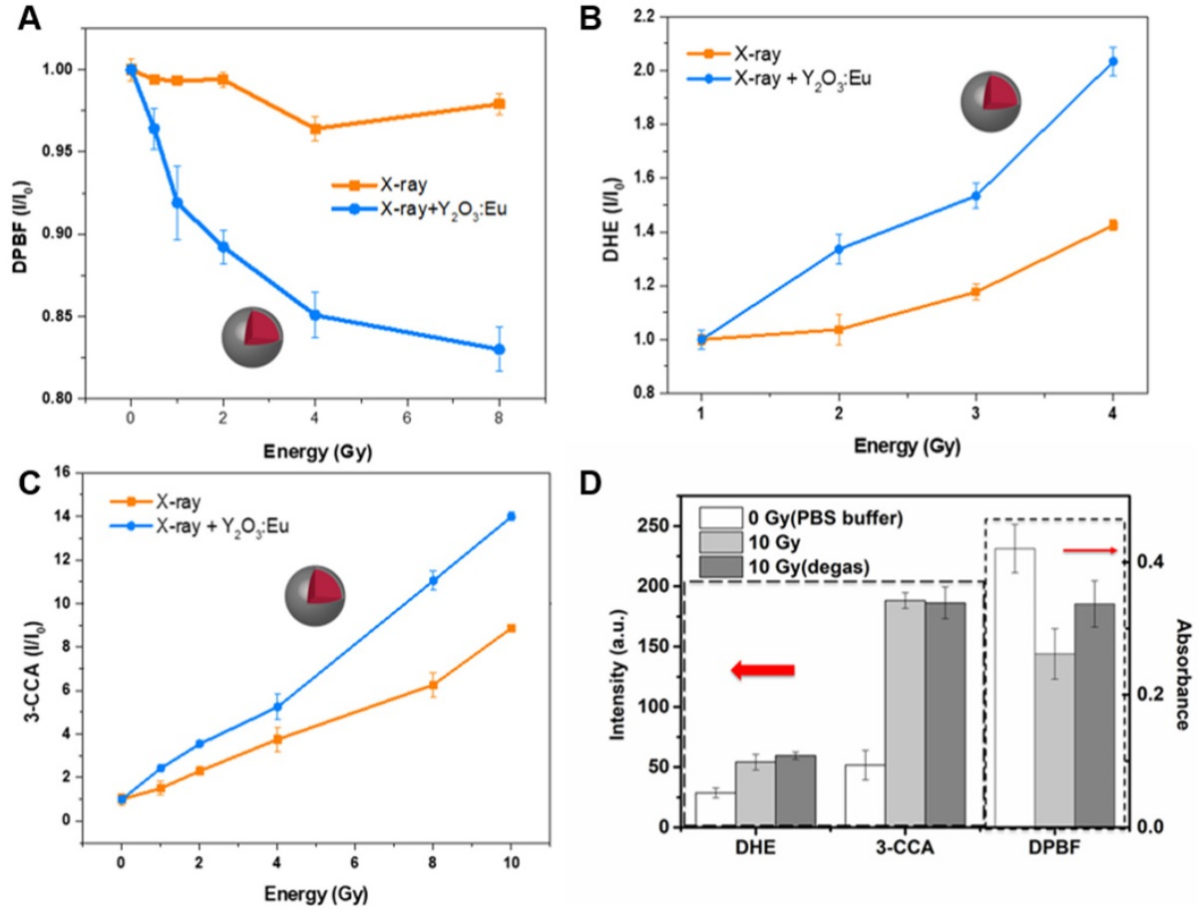
** ROS generation kinetics of Y_2_O_3_:Eu@SiO_2_ as a function of X-ray absorbed energy.** (A) ^1^O_2_ measured by DPBF; (B) O_2_^•-^ measured by DHE; and (C) •OH measured by 3-CCA. (D) ROS generation of Y_2_O_3_:Eu@SiO_2_ before (light grey) and after bubbling N_2_ gas (dark grey) with 10 Gy X-ray. The samples of Y_2_O_3_:Eu@SiO_2_ without X-ray irradiation is as control group (checkered).

PDT allows the destruction of tumors by generating more reactive oxygen species, most often by the singlet oxygen via type II reaction. Herein, the contribution of Y_2_O_3_:Eu@SiO_2_ to the enhanced generation of singlet oxygen production under X-ray irradiation was quantified by measuring the decrease in the optical absorption at 420 nm of Y_2_O_3_:Eu@SiO_2_ nanoparticles suspended in DPBF. **Figure 3A** illustrates the photobleaching of DPBF in H_2_O in the presence of Y_2_O_3_:Eu@SiO_2_ nanoparticles as a function of X-ray dose, relative to a pristine DPBF control (nanoparticle-free) solution. The decrease of DPBF absorption with X-ray dose in the Y_2_O_3_:Eu@SiO_2_ nanoparticle suspension reflects the efficient generation of highly reactive ^1^O_2_. Recently, some groups reported that silica coating enhanced luminescent intensity and suspensibility of Eu-doping phosphor 53-55. However, with a thick silica layer, the efficiency of the radio-sensitizer may be reduced because it is difficult for reactive singlet oxygen species to diffuse out of the silica layer 56. In our study, the result demonstrated that there was no obvious effect in the sensitization enhancement under thin silica coating.

Besides singlet oxygen, other biologically important reactive oxygen species (ROS), such as •OH and O_2_^•-^, were also characterized during the X-ray excitation of Y_2_O_3_:Eu@SiO_2_ nanoparticles. The DHE is highly sensitive to O_2_^•-^ and responses increased as the X-ray absorbed energy. As shown in **Figure 3B**, only Y_2_O_3_:Eu@SiO_2_ yielded a considerable amount of O_2_^•-^ under X-ray irradiation. Furthermore, we employed 3-CCA as a trapper to determine whether hydroxyl radical generation can be enhanced by irradiated Y_2_O_3_:Eu@SiO_2_. From the result shown in **Figure 3C**, one can clearly observe the enhanced generation of •OH that arises from the presence of Y_2_O_3_:Eu@SiO_2_ nanoparticles as a function X-ray irradiation dose. Furthermore, it was statistically determined that the ^1^O_2_, O_2_^•-^, and •OH generations were enhanced by 8.0, 5.4 and 1.6-fold, respectively, in use of Y_2_O_3_:Eu@SiO_2_ mediated X-ray PDT.

**Scheme 1:**
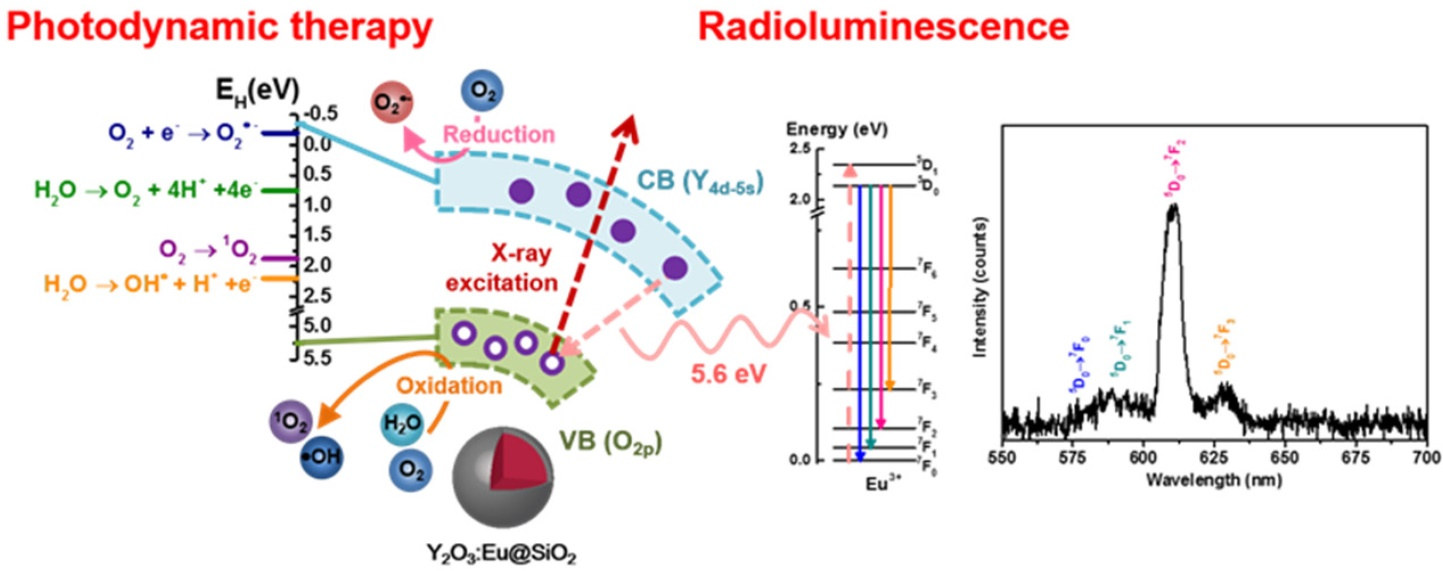
Plot for potential mechanism of ROS generation and radio-luminescence (RL) in Y_2_O_3_:Eu@SiO_2_.

In previous investigations, metallic materials with a high atomic number (Z), such as gold nanoparticles have been demonstrated to enhance photoelectric and Compton effects (and thus the subsequent emissions of secondary electrons) to augment radiation therapy efficacy. However, there are only limited investigations on ROS generation from scintillators. Y_2_O_3_ was shown to be an effective scintillator that procures self-trapped excitons at defect sites within the bulk matrix under X-ray irradiation. The incident X-ray photons promote electronic transitions from the valence band to the conduction band and create holes (h^+^) with oxidizing power in the valence band, and electrons (e^-^) with reducing power in the conduction band, respectively 57,58. In this work, the contribution of annealing-treated Y_2_O_3_:Eu@SiO_2_ to the enhanced generation of a specific type of ROS (e.g., •OH, O_2_^•-^ and ^1^O_2_) could be related to the electronic structures of the Y_2_O_3_:Eu@SiO_2_ nanoparticles, as well as the redox potentials (E_H_) of the different ROS generation reactions. Specifically, upon X-ray illumination, a part of energy could transfer from Y_2_O_3_ nanoscintillators to the Eu^3+^ optical center for fluorescence emission; another part of X-ray energy would be imparted to generate electron-hole pairs (**Scheme 1**). The photoexcited electrons and holes then react with an aqueous electron acceptor (i.e., molecular oxygen) and donor (i.e., water and hydroxyl ions), respectively, to produce different types of ROS. In a previous study, Li and coworkers reported that ROS generation reactions are thermodynamically favorable and predictable by aligning valance band (Ev), conduction band (Ec), and E_H,_ and vice versa 59. As has been demonstrated in previous research, the band gap of Y_2_O_3_ is 5.6 eV, and the theoretical Ev and Ec were 5.25 eV and -0.35 eV, respectively 60. As shown in **Figure 3**, our experimental results indicated that Y_2_O_3_:Eu@SiO_2_ generated more O_2_^•-^ than the control alone. This suggests that, since the Ec value of Y_2_O_3_ (-0.35 eV) is lower than the E_H_ of O_2_/ O_2_^•-^ (-0.2 eV), electrons could transfer from Y_2_O_3_ to O_2_ and drive superoxide anion generation. Similarly, the E_H_ for •OH and ^1^O_2_/O_2_ generation is approximately 2.2 eV and 1.8 eV, respectively. This means that the holes of Y_2_O_3_ (Ev = 5.25 eV) could theoretically oxidize H_2_O into •OH, and drive ^1^O_2_ production in Y_2_O_3_ solution. It is noteworthy that, unlike conventional PDT that is dependent on the availability of oxygen concentration for Type II reaction, our established PDT effect comprises both Type II energy transfer (oxygen-dependent) and Type I redox reactions (oxygen-independent), and thus minimizes the impediment imposed by the tumor hypoxia on PDT. To further evidence this claim and investigate forms of induced ROS, Y_2_O_3_:Eu@SiO_2_ responses to the absence of O_2_ gas were monitored using different indicators for specific ROS inductions following X-ray irradiation. As shown in **Figure 3D**, a pronounced decrease of singlet oxygen was observed after O_2_ deprivation under ambient temperature. By contrast, no significant reductions were found in hydroxyl radical and superoxide anion generations in the absence of O_2_. Given the observations, Y_2_O_3_:Eu@SiO_2_ produces comprehensive X-ray activated photodynamic reactions that encompass ^1^O_2_, hydroxyl radical, and superoxide anion, implicating its highly potential utility in PDT for hypoxic cancers.

**Figure 4:**
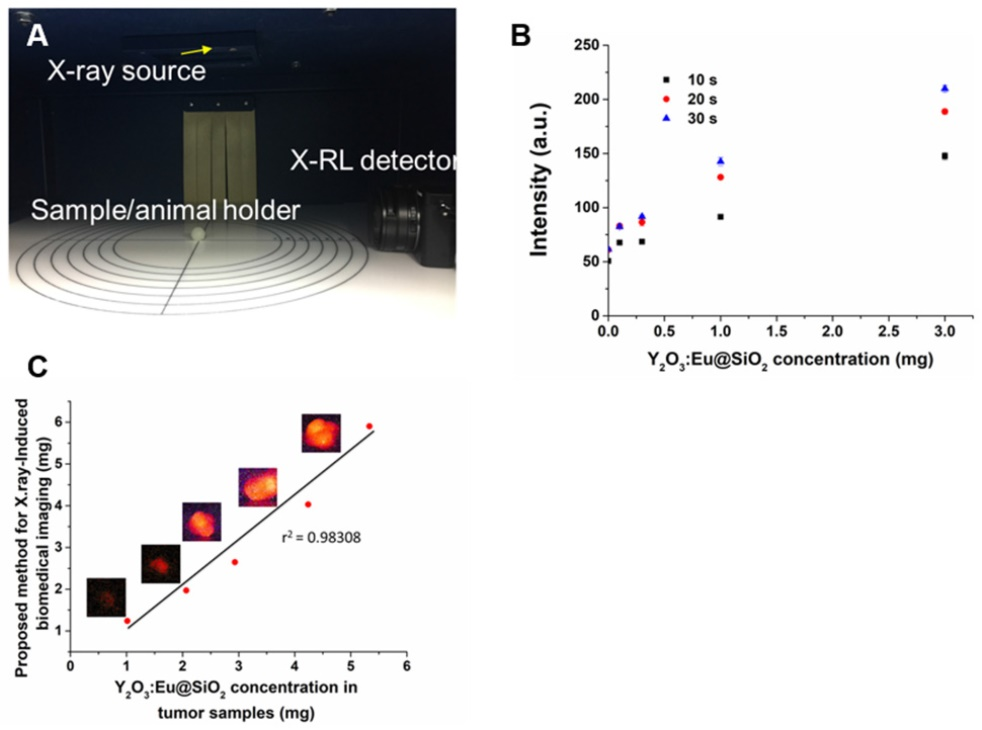
** X-RL image for deep tissue imaging activated by X-ray.** (A) X-RL imaging system consists of a highly sensitive X-RL detector (CCD camera) and X-ray irradiator cabinet is enclosed within a light tight environment. (B) Exposure sensitivity of the X-RL imaging system was assessed by exciting Y_2_O_3_:Eu@SiO_2_ phantoms under 160 kVp X-rays. (C) The in-vivo calibration curve of X-RL intensity vs. particle concentration using subcutaneously inoculated SKOV3 tumor model.

In order to investigate the use of Y_2_O_3_:Eu@SiO_2_ for biomedical X-ray excited optical luminescence (XEOL) imaging applications, a cabinet irradiator unit was outfitted with a detector positioned perpendicular to a light-tight imaging space (**Figure 4A**). To determine the temporal resolution of the X-RL imaging system, RL from 0.1 to 3 mg of Y_2_O_3_:Eu@SiO_2_ in 10% gelatin with 1% intralipid phantom were captured under various detector exposures following X-ray irradiation (**Figure 4B**). Next, to confirm the practical application of X-RL of Y_2_O_3_:Eu@SiO_2_, various concentrations of Y_2_O_3_:Eu@SiO_2_ were i.t injected into the back flanks tumor of the mice. Anesthetized mice were imaged over 10 s using our custom X-RL imaging system operating under 160 kVp and 5 mA. RL emissions from the 1 mg Y_2_O_3_:Eu@SiO_2_ inclusion were clearly detectable from the injection site (**Figure 4C**). In order to test the feasibility of our proposed method in real samples, the mice were sacrificed, and the unknown concentrations of Y_2_O_3_:Eu@SiO_2_ in different tumors were analyzed using ICP-MS. The analytical results are shown in **Table 1**. The results obtained with X-RL imaging were in good agreement with those obtained by using the ICP-MS method, with a relative error of less than 10%, except 1 mg of Y_2_O_3_:Eu@SiO_2_. These results demonstrate that our proposed method in this work is applicable for real-time detection in *in vivo* study. As shown in** Figure 3**, we have demonstrated that the ability of ROS generation of Y_2_O_3_:Eu@SiO_2_ is independent of europium dopant ion concentration. In addition, in consideration of lanthanide ions are close in ionic radius to yttrium (for example, 1.933 A° for Yb, 1.761 A° for Er and 1.8 A° for Y), the lanthanide ions could be incorporated into the Y_2_O_3_ host without significant lattice distortions. It implicated that we could replace europium with other rare-earth elements (such as Yb^3+^ and Er^3+^), so to shift the corresponding radioluminescences toward longer wavelengths for better tissue penetration.

**Figure 5:**
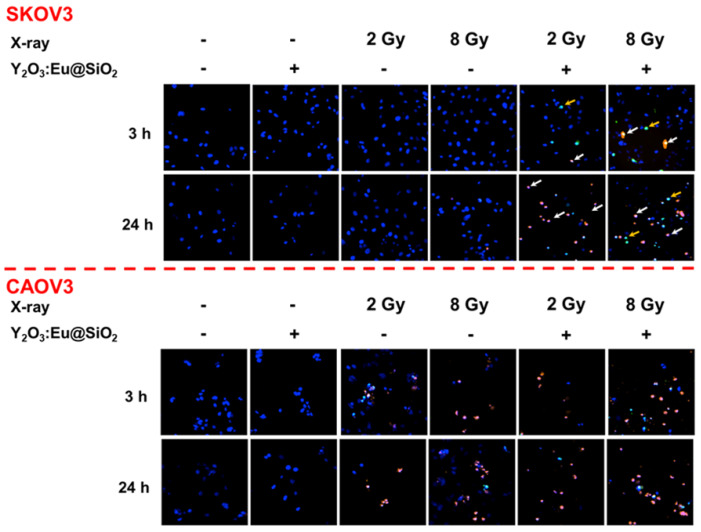
** X-ray PDT induced cellular damage in ovarian cells.** Confocal fluorescence microscopy images showing the radiotherapy performance of radioresistant SKOV3 (upper) and radiosensitive CAOV3 (bottom) cancer cells. YO-PRO-1 (green) and propidium iodide (PI) signals (red) denote leakage of the cell and nuclear membranes, respectively. The accumulative Y_2_O_3_:Eu@SiO_2_ enhanced the ROS generation and increased frequencies of early apoptosis (yellow) and necrosis (white).

Prior to any biological application, the cytotoxicity of the as-prepared Y_2_O_3_:Eu@SiO_2_ was first assessed by an MTT assay. Different concentrations of Y_2_O_3_:Eu@SiO_2_ were incubated with either CAOV3 or SKOV3 cells for 24 h in cell culture medium. As shown in **Figure S1**, at a concentration as high as 100 μg mL^-1^, the cell viability of both CAOV3 and SKOV3 still remained above 90% after 24 h incubation with Y_2_O_3_:Eu@SiO_2_, demonstrating the excellent biological compatibility of Y_2_O_3_:Eu@SiO_2_. As mentioned above, we have demonstrated that the X-ray irradiation of annealing-treated Y_2_O_3_:Eu@SiO_2_ nanoparticles generated ROS *via* comprehensive photodynamic effects (both Type I and II). Consequently, we evaluated if the ability of the as-synthesized Y_2_O_3_:Eu@SiO_2_ to generate ROS and induce cell damage after exposure to X-ray radiation which using both radio-sensitive (CAOV3) and radio-resistant (SKOV3) cell lines. As shown in **Figure 5**, untreated and Y_2_O_3_:Eu@SiO_2_ treated CAOV3 cells exhibited negligible change in cell viability. Under X-ray irradiation, cells showed positive for PI staining within 3 h, as an indication of cell death, and the number of viable cells decreased after 24 h incubation. In contrast with CAOV3 cells, SKOV3 cells exhibited negligible change in cell viability in untreated, Y_2_O_3_:Eu@SiO_2_, and X-ray alone groups. We postulate that these finding reflect that the radio-resistant SKOV3 cells eliminated the ROS by its intracellular repair capability 61. The combined treatment showed cells with a positive signal for YO-PRO-1 at the initial phase (3 h), indicating changes in plasma membrane permeability. At the late phase (24 h), the majority of cells were positive for PI staining. These results demonstrated that our Y_2_O_3_:Eu@SiO_2_ overcame the resistance of SKOV3 cells.

**Figure 6:**
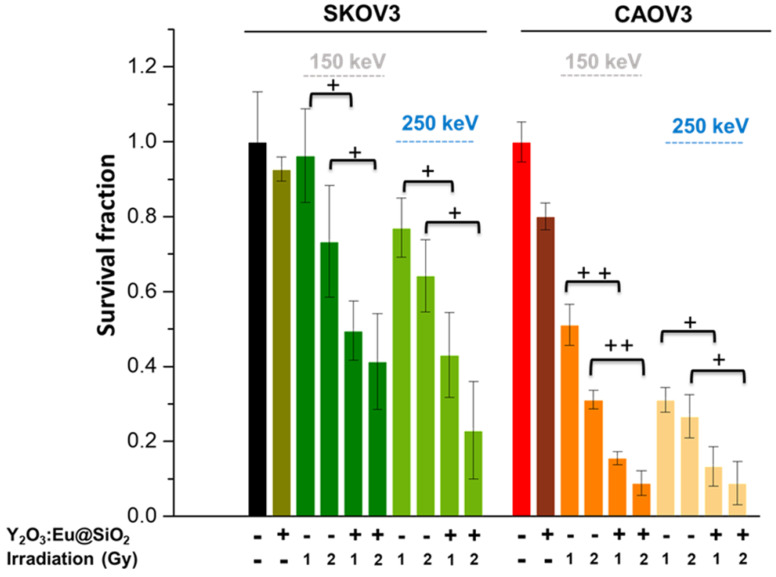
** Survival of the ovarian cells for various tube voltages.** Clonogenic survival assays of SKOV3 and CAOV3 cells treated with radiation alone or with Y_2_O_3_:Eu@SiO_2_ incubation for 24 h followed by X-ray radiation, respectively.

The destruction of tumor cells following X-ray irradiation of Y_2_O_3_:Eu@SiO_2_ nanoparticles was determined by clonogenic assay, i.e., based upon the ability of a single cell to grow, post-treatment, into a colony. As shown in **Figure 6**, significant killing of both SKOV3 and CAOV3 cells was observed in the presence of Y_2_O_3_:Eu@SiO_2_ nanoparticles with both 150 keV and 250 keV X-ray, and considerably more than with radiation alone. For the radiation-sensitive CAOV3 cells, the nanoparticles enhanced the therapeutic effect up to 40% in the 2 Gy of 150 keV X-ray. Most importantly, in the radio-resistant SKOV3 cells, more than 50% cell death was shown with Y_2_O_3_:Eu@SiO_2_ nanoparticles at 2 Gy of 150 keV X-ray irradiation. The Y_2_O_3_:Eu@SiO_2_ nanoparticles demonstrated significant therapeutic potential with lower dosage and energy X-ray irradiation in both radio-sensitive and resistant ovarian cancer cells.

**Figure 7:**
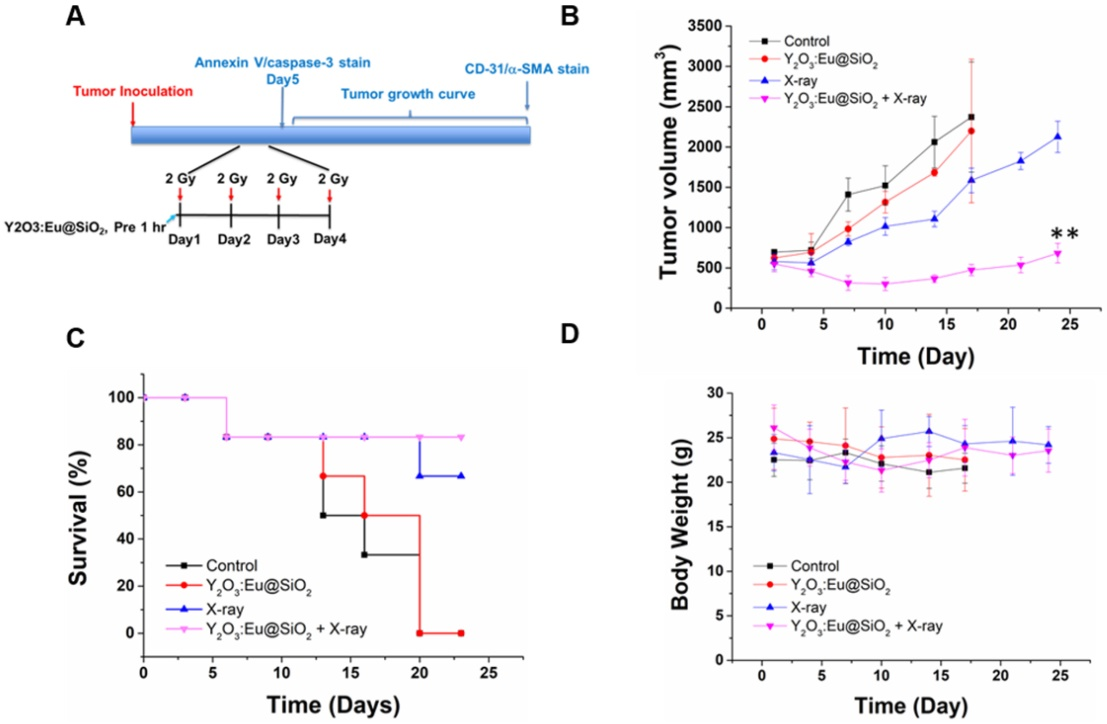
** Synergistic effects on tumor growth delay with combined X-ray PDT and fractionated radiation therapy.** (A) The treatment protocol for using hyperfractionated radiation therapy. (B) Radiosensitizing effect of Y_2_O_3_:Eu@SiO_2_ combined with X-ray irradiation in nude mice bearing SKOV3 tumors. Tumor growths after treatments with PBS, 8 Gy irradiation, Y_2_O_3_:Eu@SiO_2_ with or without 8 Gy irradiation. Each value represents the mean ± SEM of tumor volume in mm^3^ relative to that measured at the beginning of treatment. (C) Mouse survivals following various treatments. (D) The body weight of mice after various treatments.

Ovary is a radiosensitive tissue, and damage to ovary is of profound importance owing to the lack of repair of normal tissue and potentially severe sequelae. Because ovarian cancer is rarely confined to the pelvis, whole abdominal radiotherapy (WAR) was used to sterilize large volumes of micrometastatic intraperitoneal disease. Limited tolerance of the bowel, kidneys and liver also reduces the amount of radiation. Therefore, low-dose hyperfractionation could be considered as part of the standard management for ovarian cancer 62-65. Encouraged by the *in vitro* results, we further investigated an approach that is relevant to clinical stereotactic body radiation therapy: combination of radio-sensitization enhancement with a fractionated radiation regimen. Radiation was administered in four fractions of 8 Gy every other day (q1d×4) (**Figure 7A**). The effects of various treatments on the growth of subcutaneous SKOV3 tumors are shown in** Figure 7B**. The tumors in the control group progressively increased in size, which enlarged nearly by four-fold in tumor volume within 14 d. Compared with the aggravating increase tendency in the control group, either Y_2_O_3_:Eu@SiO_2_ or X-ray radiation alone caused visible tumor growth delay within the first 4 d, accompanied by an approximately 25% and 50% tumor volume decrease at the end of 14 d, respectively. In the combination regimen, the tumor growth inhibition was enhanced; resulting in tumor growth stasis during the period of 25 d. Overall, tumor volume was delayed by five-fold compared to the control (474 mm^3^ vs. 2371 mm^3^). After 24 d, five of the six mice treated with Y_2_O_3_:Eu@SiO_2_ and X-ray survived. The survival rates in those treated with X-ray alone, Y_2_O_3_:Eu@SiO_2_ alone and control group were 66%, 0%, and 0%, respectively. (**Figure 7C**). No obvious toxicity was observed in the mice following Y_2_O_3_:Eu@SiO_2_ administration, such as sudden death, abnormal behavior, significant changes of food in body weight. In addition, the histopathology examination confirmed that the structural patterns of major organs, including heart, liver, spleen, lung and kidney, of both the Y_2_O_3_:Eu@SiO_2_ combined with X-ray irradiation group and X-ray alone group are similar to those of the control group. These results suggest that no obvious acute toxicity reaction was induced in the treatments (**Figure S2**).

**Figure 8:**
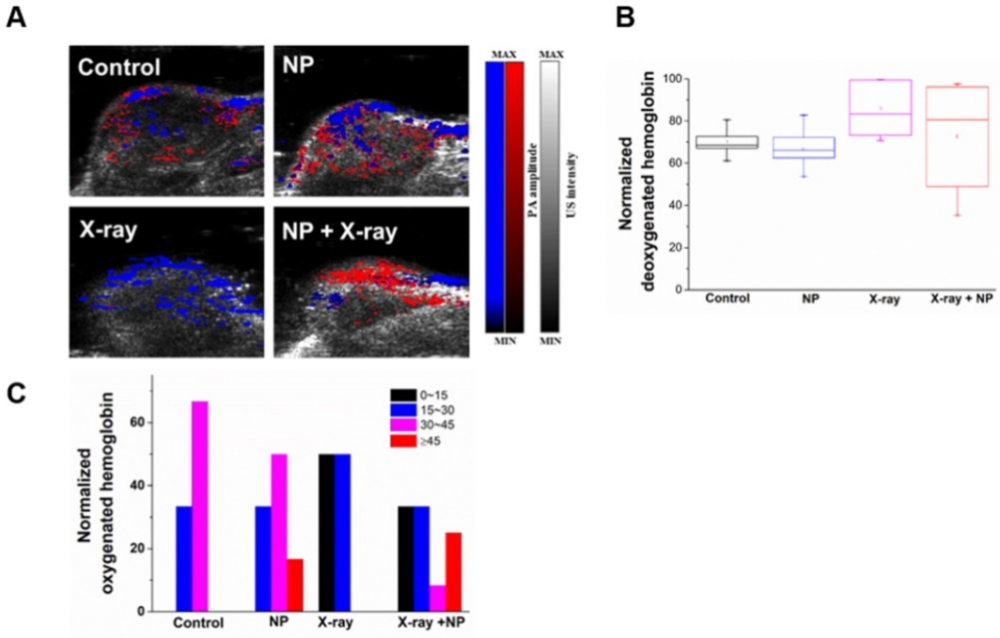
** Validation of hypoxic conditions observed in photoacoustic images *in vivo*.** (A) Oxygen saturation of a representative tumor cross-section profiled by photoacoustic image. (B) Mean deoxygenated hemoglobin of each group after X-ray irradiation. (C) Illustrations of the heterogeneous oxygenated hemoglobin distribution of different treatment groups.

It is well known that the response of tumor cells to radiation is closely related to oxygen supply through blood perfusion, and that fractionated radiotherapy minimizes radiation-induced vascular damage, thereby allowing reoxygenation of hypoxic tumor cells. To evaluate the contribution of Y_2_O_3_:Eu@SiO_2_ presence to radiation dose enhancement and reoxygenation, photoacoustic imaging (PAI) was performed to discern oxygen saturation (_S_O_2_) from the oxygenated and deoxygenated hemoglobin at different wavelengths of 850 and 750 nm, respectively 66-68. As seen in **Figure 8A**, the PAIs did not show any observable changes between the control and Y_2_O_3_:Eu@SiO_2_ alone group. Furthermore, we noticed an unusual _S_O_2_ decrease was found in the X-ray alone group after the treatment process. Surprisingly, the PAI in the combined group exhibited an observable increase in the _S_O_2_. This is of particular interest when considering potential applications of Y_2_O_3_:Eu@SiO_2_ in X-ray PDT. Through measurement of _S_O_2_ levels, the relative mean _S_O_2_ showed a slight difference between the X-ray and the combined group (**Figure 8B**). Furthermore, the normalized _s_O_2_ at the periphery of Y_2_O_3_:Eu@SiO_2_ were obtained and quantified. It was found that the tumor treated with X-ray leads to a decrease of the _S_O_2_ distribution (_s_O_2_ was 50% at _s_O_2 0~15%_ and 50% at _s_O_2 15~30%_), when compared to the control (_s_O_2_ was 33.3% at _s_O_2 15~30%_ and 66.7% at _s_O_2 30~45%_) and the Y_2_O_3_:Eu@SiO_2_ alone groups (_s_O_2_ was 33.3% at _s_O_2 15~30%_, 50% at _s_O_2 30~45%_ and 16.7% at _s_O_2 ≥45%_) (**Figure 8C**). This phenomenon is in agreement with the extant literature 69, and is ascribed to endothelial death (with a threshold dose of 5~10 Gy) and a decrease in oxygen supply after X-ray irradiation. Moreover, Hasan et al. reported that PDT, as well as high single-dose radiation therapy, can create a temporal hypoxia region in tissue adjacent to perfused blood vessel 70. This event might be expected to attenuate radiation therapy response. Additionally, an inadequate supply of O_2_ (hypoxia) has long been recognized as a cause of radio-resistance, in which hypoxic tumor cells are approximately three times more resistant to radiotherapy (known as ''the oxygen effect”). Interestingly, the Y_2_O_3_:Eu@SiO_2_ with X-ray group showed enhanced _s_O_2_ level at the periphery of tumor administered with Y_2_O_3_:Eu@SiO_2_ (_s_O_2_ was 33.3% at _s_O_2 0~15%_, 33.3% at _s_O_2_ 15~30%, 8.4% at _s_O_2 30~40%_ and 25% at _s_O_2 ≥45%_). It could be reasonably speculate that, upon X-ray PDT, the oxygen demand in tumors is drastically diminished due to severe damage to tumor cells. On the other hand, the oxygen supply through blood perfusion continuously contributes to the intratumoral oxygen tension. Potiron et al. demonstrated that fractionated radiotherapy increased intratumoral doxorubicin diffusion 71. These results are in accordance with Hansen et al., who reported that tumors displayed an increased mean accumulation of liposomes for radiation doses up to 10 Gy 72. We observed obvious difference between the _s_O_2_ values of the X-ray alone and the combined groups at 1 h post-X-ray PDT.

**Figure 9:**
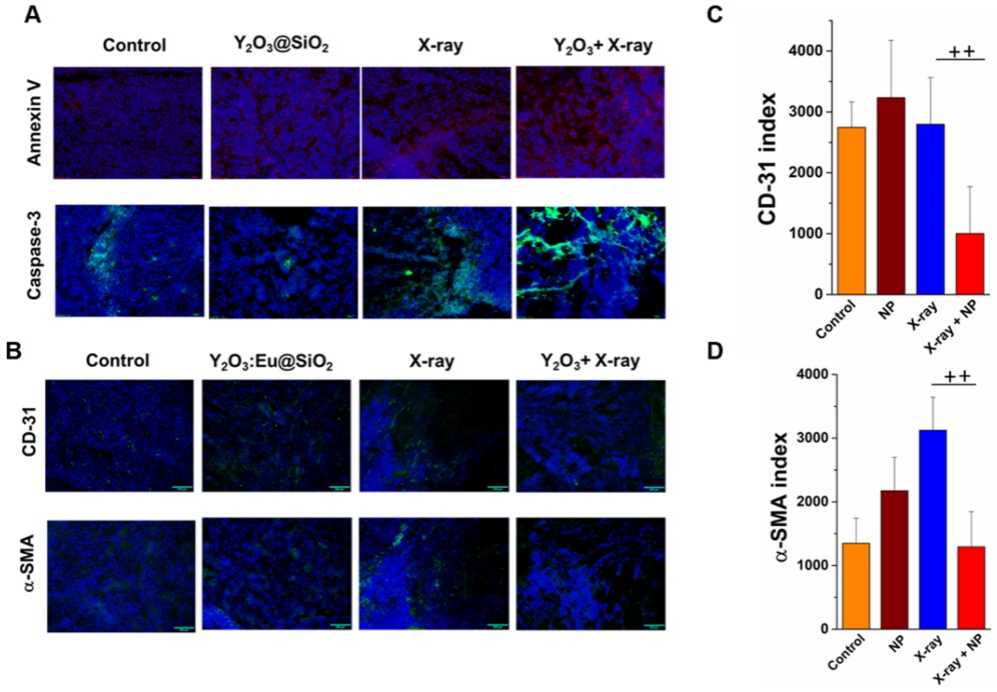
** RT induces tumor redistribution and vascular damage.** (A) Representative scanner images of immunohistochemistry for Annexin V and caspase-3 in SKOV3 tumors. (B) Representative scanner images of immunohistochemistry for endothelial cells (CD-31) and pericytes (α-SMA) for the different treatment groups after the four days radiation therapy. (C) (D) quantification of CD-31^+^ and α-SMA in SKOV3 tumor. Index values shown in (C) and (D) represent the mean of n≥3 ± SEM.

Induction of apoptosis in SKOV3 tumors at the end after various treatments is depicted in **Figure 9A**. As illustrated in **Figure 9A**, tumors treated with X-ray combined with Y_2_O_3_:Eu@SiO_2_ showed the highest Annexin V expression, an early apoptosis marker, as compared to tumors with only X-ray irradiation and with Y_2_O_3_:Eu@SiO_2_ alone. Moreover, the strongest caspase 3 signal was also observed in the group of X-ray combined with Y_2_O_3_:Eu@SiO_2_, suggesting that Y_2_O_3_:Eu@SiO_2_ effectively enhanced radiotherapy *via* cell apoptotic cascade. To explore other possible mechanisms besides apoptosis that synergistically contributed to the enhanced inhibition of tumor growth in the presence of our annealed Y_2_O_3_:Eu@SiO_2_, we further assessed tumors harvested from various groups with immunofluorescence (IF) imaging of CD-31. The IF images showed a decrease in CD-31 positive endothelial cells at the end of treatment compared to the other groups (**Figure 9B**). Consistent with previous reports, high doses of radiation induced additional tumor cell killing through “non-classical” radiobiological mechanisms, mediated by tumor microvascular damage 73-75. Specifically, Demidov et al. carried out longitudinal* in vivo* imaging observations, and demonstrated that maximal tumor shrinkage increases with dose, and the time to initial vascular volume density recovery is also dose-dependent. In our study, _s_O_2_ in the combined group increases rapidly after irradiation, probably due to damage in the endothelial cells followed by widening of the gaps between endothelial cells. However, we also observed the vascular volume density declined at the end of treatment in combined group. Wang and colleagues reported copper-cysteamine NPs-mediated X-PDT, which caused a decrease in the number of vessels, but did not inhibit the growth of radio-resistant melanoma 76. In our study, we demonstrated NP-mediated X-ray PDT combined with fractionated radiation therapy, which decreased the tumor vessels at the end of *in vivo* study. Further, multi-shots X-ray PDT treatment improved oxygen distribution at the periphery of tumor administered with Y_2_O_3_:Eu@SiO_2_, and enhanced efficiency of X-ray to inhibit the growth of radio-resistant SKOV3. To further validate and assessed these findings, CD-31 and α-smooth muscle actin (α-SMA) stain was utilized to depict the vascular remodeling and pericyte recruitment. As shown in **Figure 9C**, a decrease in CD-31 (64%) intensity was found at the end of treatment compared to non-irradiated and NP alone tumors. Interestingly, in the X-ray alone group, an increase in α-SMA intensity was observed after three weeks of RT compared to non-irradiated (2.3-fold), NP alone (1.4-fold), and NPs combined with X-ray irradiated tumors (2.4-fold), indicating that the sublethal dose of irradiation could enhance radiotherapy-induced vascular remodeling at the late stage (**Figure 9D**). The X-ray induced vascular remodeling indexed with α-SMA was compromised in combined group is probably ascribed to the ROS generated by the annealed Y_2_O_3_:Eu@SiO_2_ following X-ray irradiation, where the photoexcited electrons and holes of Y_2_O_3_ react with an aqueous electron acceptor (i.e., molecular oxygen) and donor (i.e., water and hydroxyl ions) to produce different types of ROS that abolish pericyte recruitment. Together, these findings implicate that annealed Y_2_O_3_:Eu@SiO_2_ combined with fractionated radiation therapy has the potential to be utilized to reverse vascular remodeling-enhanced tumor resistance to radiation therapy.

## Conclusion

In summary, we have developed Y_2_O_3_:Eu@SiO_2_ nanoscintillators that could be potentially used for X-RL imaging-guided radiotherapy with a significant radio-sensitization enhancement. It is notable that the annealing pretreatment is critical for Y_2_O_3_:Eu@SiO_2_ to render such a PS-independent radio-sensitization enhancement that comprises comprehensive photodynamic effects and pronounced RL for possible imaging guidance and *in-situ* dosimetry assessment. This report further demonstrated that RL emission and ROS generation constitute two parallel and independent mechanisms, which is important as it implicates that the Y_2_O_3_:Eu@SiO_2_ could harness the ionizing radiation efficiently to reach the maximal theranostic utilization. In imaging, the red-emitted X-RL of Y_2_O_3_:Eu@SiO_2_ was quantitatively monitored for nanoparticle tumor accumulation and dosimeter assessment *in vivo*. However, in consideration of the penetration depth and scattering attribute of red X-RL in tissue, the quantitative dosimetry and imaging of Y_2_O_3_:Eu@SiO_2_ are constrained to superficial tumors. We have demonstrated the ability of ROS generation of Y_2_O_3_:Eu@SiO_2_ is independent of europium dopant ion concentration. Furthermore, due to the similar ionic radius of lanthanide ions to yttrium (for example, 1.933 A° for Yb, 1.761 A° for Er and 1.8 A° for Y), the challenge of producing high-penetration X-RL in our nanotheranostics may be addressed by substituting Eu^3+^ with other rare-earth-ions such as Yb^3+^ and Er^3+^ in the doping process, to possibly tuning and extending the peak emission wavelengths to near infrared I and II regions. In addition, the generation of electron-hole pairs in Y_2_O_3_ could react with an aqueous electron acceptor (i.e., molecular oxygen) and donor (i.e., water and hydroxyl ions), which essentially minimize oxygen dependency for the generation of reactive oxygen species. Furthermore, for the *in-vivo* radio-sensitizing effect of our established nanoparticles, the preliminary results from tumor xenografts suggested that the therapeutic enhancement observed *in vivo* by Y_2_O_3_:Eu@SiO_2_-derived radio-sensitization could be readily translated to favorable radiotherapy for tumor control. A new indication is noteworthy that Y_2_O_3_:Eu@SiO_2_ in combination with fractionated radiation therapy could induce vascular remodeling that accompanied by decreased hypoxia distribution intratumorally. It is anticipated to alleviate the radiobiological effect associated with tumor radioresistance and to substantially improve conventional PDT.

## Supplementary Material

Supplementary figures and tables.

## Figures and Tables

**Table 1:** Comparison of Y2O3:Eu@SiO2 concentrations measured using ICP-MS and X-RL in subcutaneously inoculated SKOV3 tumor model

Sample No.	ICP-MS	Proposed method^a^	Relative error (%)
1	1.0158 (mg)	1.2393 (mg)	18%
2	2.0641 (mg)	1.9695 (mg)	4.8%
3	2.9323 (mg)	2.6457 (mg)	10.8%
4	4.2409 (mg)	4.0297 (mg)	5.2%
5	5.3345 (mg)	5.9025 (mg)	9.6%

a Using radioluminescence property of Y_2_O_3_:Eu@SiO_2_.
