# Supplementary material for: Annealing-modulated nanoscintillators for nonconventional X-ray activation of comprehensive photodynamic effects in deep cancer theranostics
Source: Theranostics. 2020 May 20;10(15):6758–73. doi: 10.7150/thno.41752 (PMC7295068; doi:10.7150/thno.41752)
Supplement: Supplementary file 1 — Supplementary figures and tables. [file thnov10p6758s1.pdf]

Supplementary data for

Annealing-modulated nanoscintillators for nonconventional X-ray activation of comprehensive photodynamic effects in deep cancer theranostics

Yao-Chen Chuang,[§ a] Chia-Hui Chu,[§ a] Shih-Hsun Cheng,[a, b] Lun-De Liao,[a] Tsung-Sheng Chu,[a] Nai-Tzu Chen,[c] Arthur Paldino,[d] Yu Hsia,[a] Chin-Tu Chen,[b] and Leu-Wei Lo[*,a]

[a] *Institute of Biomedical Engineering and Nanomedicine Research, National Health Research Institutes, 35 Keyan Road, Zhunan 350, Taiwan.*

[b] *Department of Radiology, The University of Chicago, Chicago, IL 60637, USA.*

[c] *Department of Cosmeceutics, China Medical Univsesity,91 Hsueh-Shih Road, Taichung, Taiwan 40402*

[d] *Faculté de médecine Lyon-Est, Université Claude Bernard, 8 Avenue Rockefeller, Lyon 69008, France*

[§] These authors contributed equally

* Author for correspondence:

Leu-Wei Lo, Ph.D.

Institute of Biomedical Engineering and Nanomedicine, National Health Research Institutes, Zhunan, Miaoli 35053, Taiwan.

Tel: 886-37-246166 # 37115 Fax: 886-37-586440

E-mail: lwlo@nhri.edu.tw

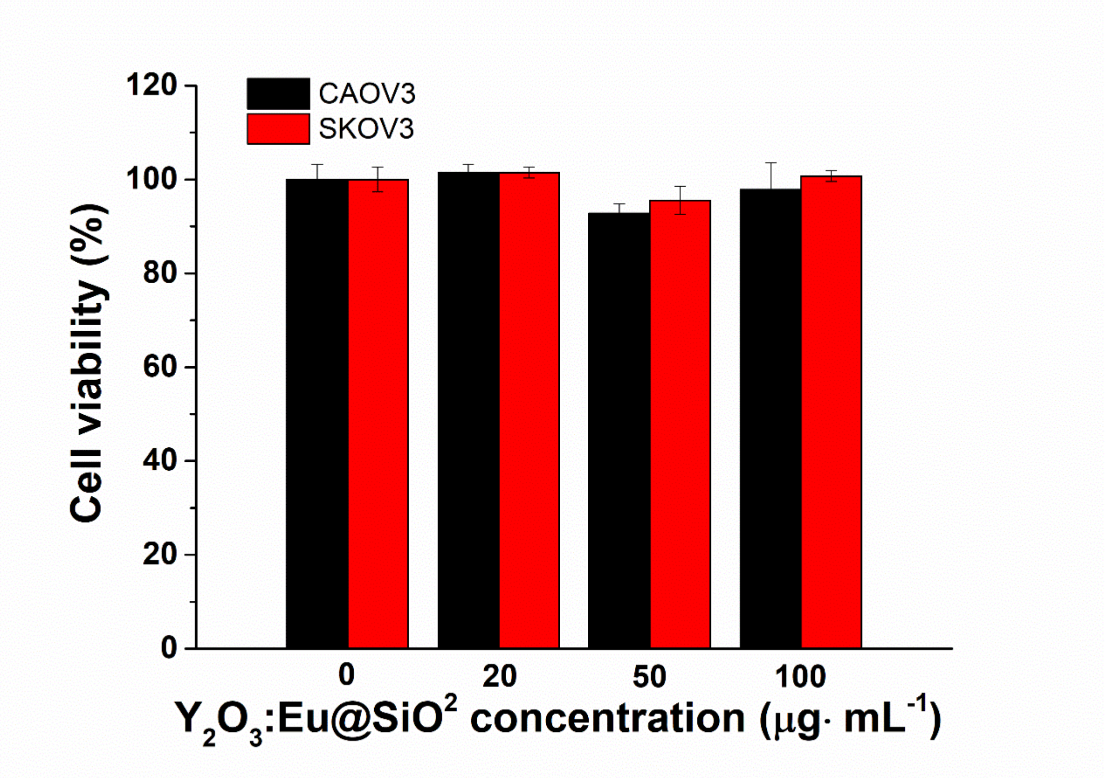

Figure S1. The survival rates of cells were determined by the MTT method after treated by 0, 20, 50, or 100 μg·mL^{-1} of Y_2O_3:Eu@SiO_2 nanoparticles for 24 h in vitro. No obvious toxicity of Y_2O_3:Eu@SiO_2 nanoparticles is observed in both CAOV3 and SKOV3 cells.

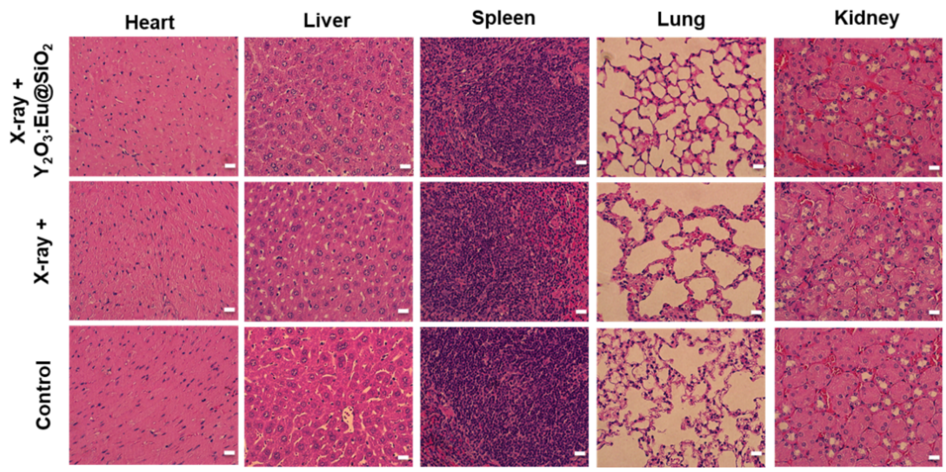

Figure S2. Histopathologic examination of the major organs collected of nu mice in the X-ray PDT group, fractionated radiation therapy group and the control group at the experimental endpoint (scale bar: 20 mm).
